# From nature experience to pro-conservation action: How generational amnesia and declining nature-relatedness shape behaviour intentions of adolescents and adults

**DOI:** 10.1007/s13280-025-02135-7

**Published:** 2025-03-06

**Authors:** Tanja M. Straka, Carolin Glahe, Ulrike Dietrich, Miriam Bui, Ingo Kowarik

**Affiliations:** 1 Department of Ecology, Technische Universität Berlin, 12165 Berlin, Germany; 2 Berlin-Brandenburg Institute of Advanced Biodiversity Research (BBIB), 14195 Berlin, Germany; 3 Institute of Biology, Freie Universität Berlin, 14195 Berlin, Germany; 4 Chair of Decarbonization and Transformation of Industry, Brandenburg University of Technology Cottbus -Senftenberg, 03046 Cottbus, Germany

**Keywords:** Biodiversity conservation, Extinction of experience, Generational amnesia, Nature-relatedness, Pro-conservation behaviour, Species identification

## Abstract

Addressing the global biodiversity crisis requires conservation engagement across generations. However, *generational amnesia* and *extinction of experience* probably threaten this effort, reflecting declines in nature knowledge, experience and engagement among younger generations. We examined urban green area visit frequency, nature-relatedness, identification skills and familiarity with species (plants, birds and butterflies) and pro-conservation behaviour intentions in adolescents (15–17 years, mean = 15.97), young adults (18–29 years, mean = 23.72) and older adults (> 30 years, mean = 40.54) in Germany. With a path analysis, we explored relationships between concepts across all age groups. Although all age groups visited green areas similarly, identification skills, nature-relatedness and intentions declined progressively from older to younger groups. Nature-relatedness predicted intentions and identification skills predicted nature-relatedness across all groups. Our findings support the importance of fostering nature-relatedness and species knowledge through age-sensitive access to urban green areas with opportunities to connect with and learn about nature.

**Supplementary Information:**

The online version contains supplementary material available at 10.1007/s13280-025-02135-7.

## Introduction

### Generational amnesia, extinction of experience and pro-conservation behaviour

Counteracting the accelerating global biodiversity crisis increasingly requires active engagement in conservation activities from both current and future generations (Ceballos et al. 2017). Enhancing people’s connection to nature is generally understood as a highly promising pathway to pro-environmental behaviour (Whitburn et al. 2020). However, there is rising concern about a trend towards disconnecting from nature, particularly in economically developed countries, potentially diminishing pro-conservation behaviour in younger generations (Jones et al. 2020; Soga and Gaston 2016, 2023; Gaston and Soga 2020). Disconnection from nature has many dimensions (Ives et al. 2018; Beery et al. 2023), including the phenomena of *generational amnesia* and *extinction of experience*. Generational amnesia is expressed as the decline of knowledge about species and as one form of shifting baselines where knowledge extinction occurs because younger generations are not aware of past biological conditions (Pauly 1995; Kahn Jr 2002; Gerl et al. 2021; Papworth et al. 2009). Accordingly, species knowledge has been found to increase with age (Hooykaas et al. 2019; Gerl et al. 2021; Enzensberger et al. 2022).

The increasing global urbanization could accelerate this trend due to the partial or complete transformation of natural landscapes and altered species communities (Elmqvist et al. 2021; Hahs et al. 2023), leading to different experiences and knowledge of species. For instance, a study in Israel showed that urban residents exhibited lower identification skills compared to their rural counterparts (Bashan et al. 2021). This could pose challenges for future engagement in conservation activities, as historical baselines remain relevant according to a global expert survey, but adjusting them to a changing world may be necessary (Clement et al. 2023). Some authors argue that if younger generations do not acknowledge the change in the status of biodiversity, they may be less engaged in conservation (Papworth et al. 2009). Accordingly, recent studies found a direct link between knowledge about species and willingness to take action for their protection (Jones et al. 2020; Enzensberger et al. 2022).

The extinction of experience is characterized by fewer human–nature interactions (that can be positive, neutral or negative) which is linked to a lower connection to nature (Pyle 1978; Miller 2005; Soga and Gaston 2016, 2020). This trend is assumed to be driven by an interplay of decreasing opportunities and orientation, with nature exposure playing a crucial role (Lin et al. 2014; Soga and Gaston 2016, 2020). Opportunities to experience nature are supposed to diminish due to accelerating urbanization and consequently, spatial barriers to access nature, altered species richness and modern lifestyle since many people are spending more time indoors and may spend less time in nature (Hartig and Kahn 2016; Soga and Gaston 2016, Gaston and Soga 2020). Despite these barriers, orientation towards nature has been identified as an even stronger driver of park visits than mere opportunity (Lin et al. 2014). The increased concern of parents for the safety of their children when playing outdoors may also contribute to this trend (Orr 2002). However, the extinction of experience among children is also controversially discussed (Oh et al. 2020; Novotný et al. 2021. When comparing a recent survey with a survey from 120 years ago, Novotný et al. (2021) found that children are much more involved in outdoor activities today than before. Yet this study did not explore changes in the orientation of children towards nature.

Scholars have raised concerns about generational amnesia and the extinction of experience since these phenomena could induce a generational shift, leading to diminished effort in behaviour concerning the environment and conservation (Papworth et al. 2009; Soga and Gaston 2016). However, comparative studies to understand whether such a shift is occurring across different age categories remain scarce to date. These studies should particularly focus on how opportunities and orientations are related to pro-conservation behaviour.

### Causes and consequences: opportunity, orientation and pro-conservation behaviour intentions

Why are opportunity and orientation (following Soga and Gaston 2016) not only important when it comes to the extinction of experience phenomena, but also for pro-conservation behaviour? First, opportunities to experience and a positive orientation for nature may be crucial to foster care for nature. A body of literature supports the importance of direct experience with nature on pro-conservation behaviour (Martin et al. 2020; Gaston and Soga 2020; DeVille et al. 2021; Soga and Gaston 2024). For instance, visiting nature more than once a week was positively associated with general health and household pro-environmental behaviours among residents in the UK (Martin et al. 2020). Further, a recent study from Japan showed that direct experiences with nature positively predicted self-reported pro-conservation behaviours (Soga and Gaston 2023). An Australian study indicates the importance of direct interactions with nature in contrast to exposure to nature: While activities performed in urban parks or on the beach were related to people’s support for conservation, the distance to urban green space, as an indicator of exposure to nature, was not (Dean et al. 2019). In this context, orientation serves as major driver for engaging with nature (Lin et al. 2014).

Nonetheless, solely having the opportunity to experience nature may not lead directly into pro-conservation behaviours. Soga and Gaston (2024) identified in their meta-analysis a link between nature experiences during child- and adulthood and pro-conservation behaviour, particularly pro-biodiversity behaviour. They emphasized that this positive association does not necessarily indicate a causal relationship between direct experiences of nature and pro-conservation behaviour. Here, they suggest individual’s emotional connection to or knowledge about nature as a pathway. Indeed, it has been suggested also by other scholars that the link between nature exposure and pro-conservation behaviour may not be direct, but moderated by concepts such as environmental knowledge and nature-relatedness (Otto and Pensini 2017). However, similarly here, a body of literature emphasizes that knowledge about nature and environmental issues alone might not lead to action unless individuals experience nature and feel emotionally connected, making environmental issues more personally relevant and motivating action (Kaplan et al. 1998; Miller 2005; Otto and Pensini 2017); as well as environmental identity and other psychological factors (Clayton and Myers 2015); although these are not covered in this study.

Nature-relatedness is one measure to assess the affective cognitive and experiential aspects of one’s connection to nature (Nisbet et al. 2009) being characterized by feelings of empathy, awe and respect for nature, as well as a sense of oneness with the natural world. Nature-relatedness plays an important role in subjective well-being, can convey feelings of happiness and is considered a fundamental motivational basis for people to protect nature (Mayer and Frantz 2004; Nisbet 2011; Nisbet and Zelenski 2023). The positive link between nature-relatedness and pro-conservation behaviour in various contexts is well established (Gosling and Williams 2010; Geng et al. 2015; Barrera-Hernández et al. 2020). This is where, secondly, orientation becomes an important aspect, not only in motivating visits to green spaces, but also in fostering for pro-conservation behaviour.

In our study, we define orientation’ more specifically than the broader terms used by Gaston and Soga (2020), who associated it with general attitudes. We understand the term to encompass various components, such as knowledge about nature, species or related issues and nature-relatedness. Although these two components are distinct, they likely influence and reinforce each other. Considering these interconnected components, a relevant question arises: when examining the link between nature experiences and pro-conservation behaviour, which component plays a more pivotal role irrespective of age? Is it knowledge or nature-relatedness?

## Theoretical Framework

Based on this background and our interest in exploring the relationship between nature exposure, knowledge about nature, nature-relatedness and pro-conservation behaviour, we built on the competence model of education (Roczen et al. 2014). This model postulates the importance of nature-relatedness and knowledge as links between nature exposure and pro-conservation behaviour (Roczen et al. 2014). Environmental knowledge and nature-relatedness are considered as related, yet independent constructs (Otto and Pensini 2017). People who feel a strong nature-relatedness may have a heightened interest in learning about it. Conversely, learning about nature can foster a deeper nature-relatedness. Both constructs are weakly interrelated, and it is unclear which comes typically first or if there is a sequential relationship at all (Otto and Pensini 2017). Empirical research on this model was undertaken to our knowledge only with schoolchildren, showing consistently that nature-relatedness was a stronger driver for pro-conservation behaviour than environmental knowledge (Roczen et al. 2014; Otto and Pensini 2017). However, to our knowledge, studies covering different age categories are missing here.

**Fig. 1:**
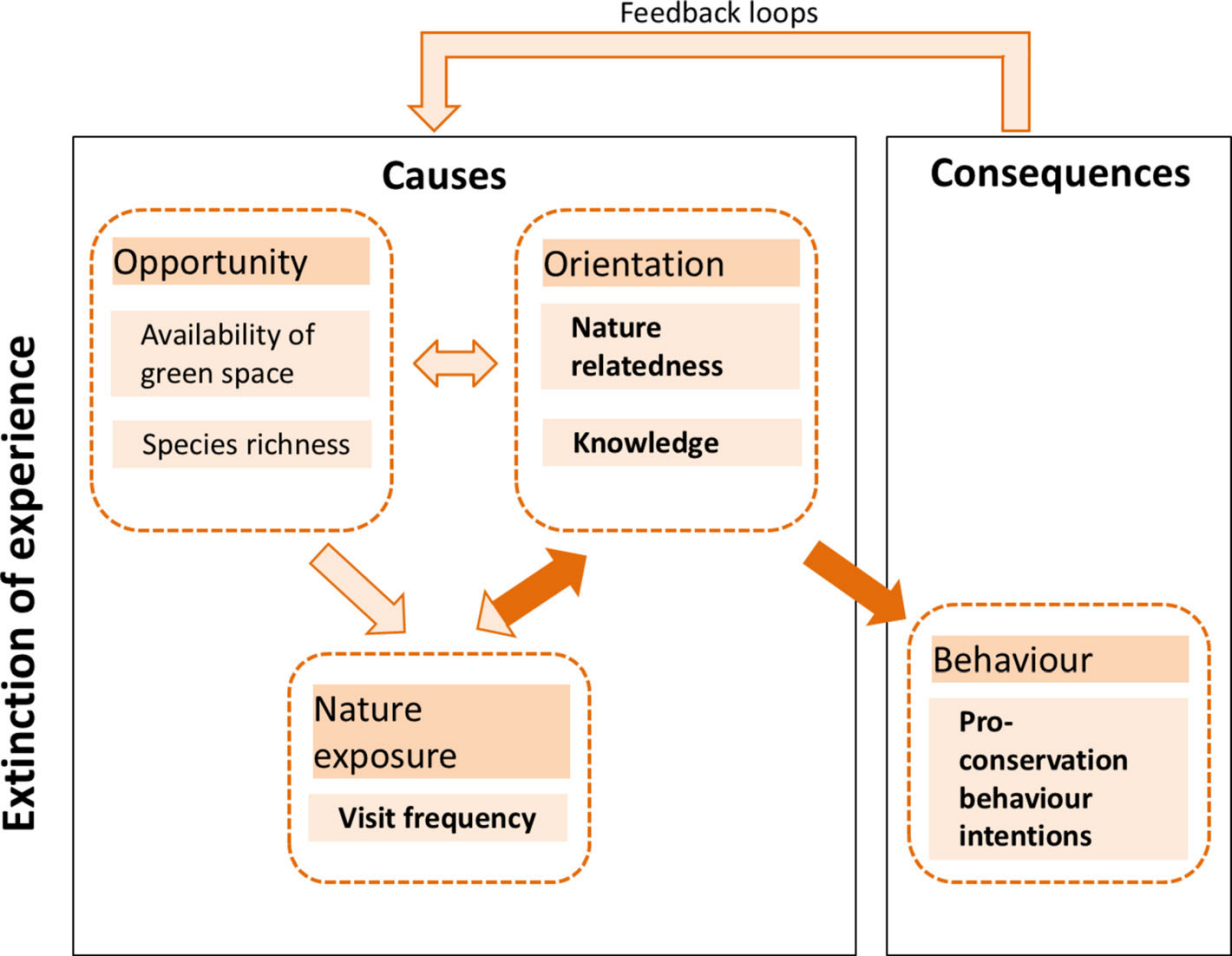
Framework showing the investigated relationships from visit frequency to pro-conservation behaviour intentions in adolescents, young and older adults; adapted from Otto and Pensini (2017) and Gaston and Soga (2016). The relationships assessed in this study are represented by dark orange arrows, while those not assessed are indicated by light orange arrows. Generational amnesia is considered within ‘causes’ as the decline of knowledge about species (Papworth et al. 2009). While opportunity is also an important component of this framework, this was not part of our study. We acknowledge that this framework has the potential to function bidirectionally, but our focus in this study is on the unidirectional path

We integrated Roczen’s (2014) competence model of environmental education with Soga and Gaston’s (2016) model on causes (i.e. opportunity and orientation) and consequences (i.e. behavioural change) of the extinction of experience phenomenon (Fig. 1). In the latter model, the frequency of contact with nature or visits to urban parks is also related to both opportunities and orientation.

In this comparative analysis of adolescents, young and older adults, we focussed on self-reported frequency of visiting urban green areas and orientation as important components of causes. Orientation towards nature was measured as species knowledge and nature-relatedness (for the latter see Colléony et al. 2020a, b). We measured species knowledge by species identification skills, a valid predictor for in-depth knowledge about species (Hooykaas et al. 2022). We further assessed in this context familiarity with species to examine the relationship between mere recognition (familiarity) and accurate identification within our study population (Bashan et al. 2021). We measured participant’s nature-relatedness using a shortened version of Nisbet and Zelenski’s (2013) NR6 scale. As for pro-conservation behaviour, we focussed on pro-conservation behaviour intentions which, according to Ajzen’s theory of planned behaviour (Ajzen 1991), represent an immediate antecedent of behaviour. However, intentions are also considered as a distinct concept that moderately correlates with behaviour (Whitburn et al. 2019).

This approach allowed us to test the relative importance of species knowledge versus nature-relatedness as moderators between visit frequency and pro-conservation behaviour intentions following the framework of Otto and Pensini (2017) across different age categories (i.e. adolescents, young and older adults, Fig. 1). It also situated our study within the ongoing debate surrounding the phenomena of generational amnesia (Papworth et al. 2009) and extinction of experience (Soga and Gaston 2016).

Understanding changes in people’s exposure to nature and its relationships with the concepts of knowledge, nature-relatedness and pro-conservation behaviour is challenging because previous studies mostly focussed on adults (Whitburn et al.^2020^), with few exceptions (Roczen et al. 2014; Collado et al. 2015; Otto and Pensini 2017; Hughes et al. 2018). Since corresponding comparisons between age categories in cross-sectional studies are lacking thus far, we performed surveys, covering adolescents (15–17 years), young (18–29 years) and older adults (> 30 years). Our study aimed to

1. Compare the potential causes in the context of the extinction of experience, namely levels of visit frequency to urban green areas, species knowledge and nature-relatedness and the possible consequences, namely pro-conservation behaviour intentions among adolescents, young and older adults.
2. Explore the roles of species knowledge and nature-relatedness (orientation) as path variables linking visit frequency to pro-conservation behaviour intentions within the adolescent, young and older adult groups.

Drawing on the extinction of experience and generational amnesia phenomena, we anticipated that younger people would exhibit lower levels of visit frequency, nature-relatedness and species knowledge compared to older people. Consequently, we expected a corresponding decrease in pro-conservation behaviour intentions from older adults to adolescents. Further, based on earlier studies that showed the role of nature-relatedness for pro-conservation behaviour intentions (Gosling and Williams 2010; Geng et al. 2015; Barrera-Hernández et al. 2020), we anticipated that nature-relatedness would have a stronger effect on pro-conservation behaviour than species knowledge. However, to our knowledge, it has not been sufficiently addressed whether nature-relatedness or species knowledge serves as stronger path variables between nature experiences and conservation behaviour and particularly, how these relationships differ across age categories. Therefore, in this study, we respond to the call for more empirical research to fully understand how nature experiences at different stages of life can shape individuals pro-environmental attitudes and behaviours (Soga and Gaston 2024).

## Materials and methods

### Development of the survey instrument

**Fig. 2:**
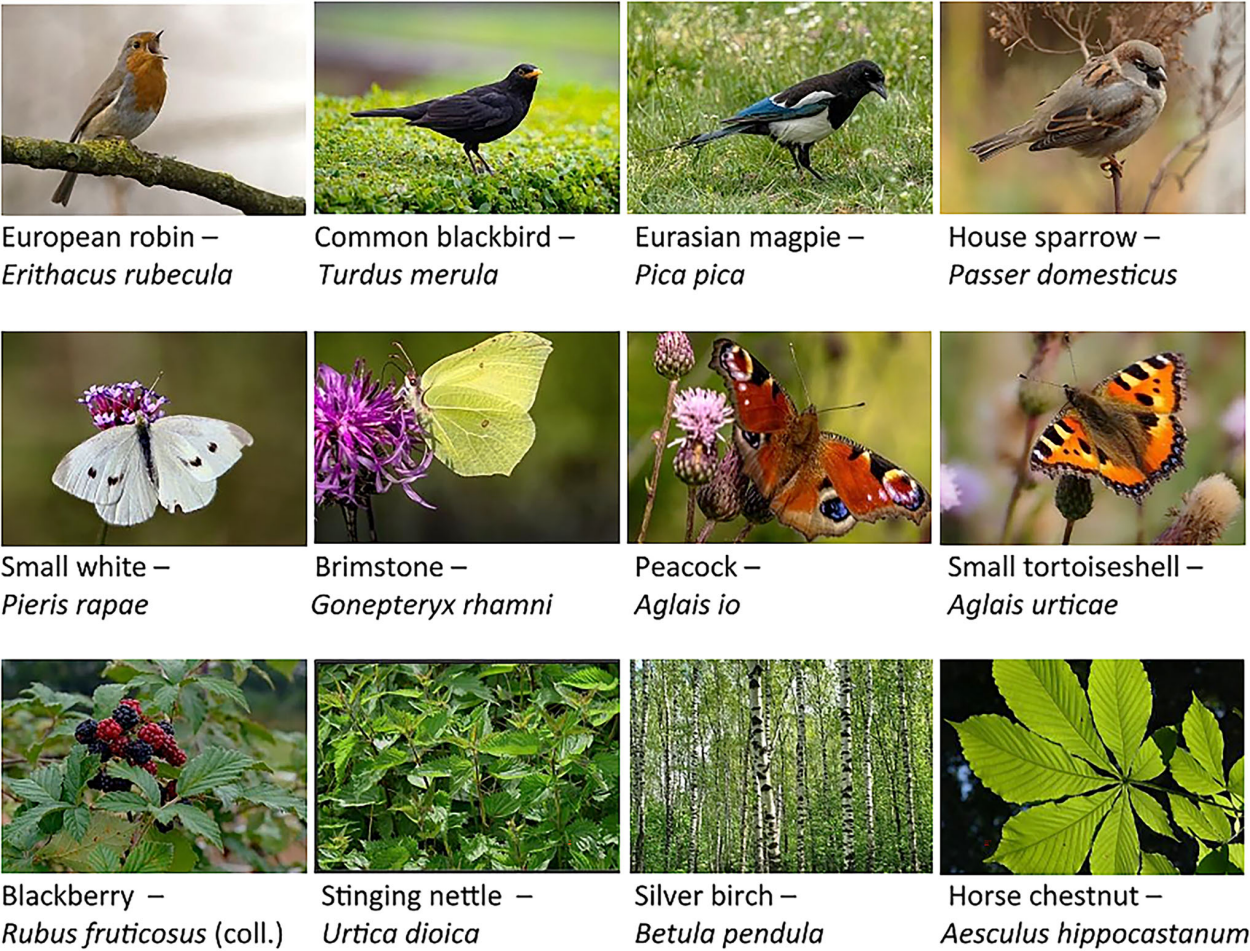
Photographs of species depicted in the surveys. (Open source photographs by Unsplash, Shutterstock, Pexels)

The online survey that adolescents and adults received consisted of four sections, following a short introduction (Table 1). The first section involved questioning participants about their familiarity with common plant and animal species, followed by an invitation to write down the names of each species depicted in the photographs. Here, we followed Bashan et al. (2021) concerning familiarity and species identification skills in relation to three groups of plants and animals. In detail, we showed participants 12 photographs, featuring four species each of birds, butterflies and plants (Fig. 2). We also selected very common and easily distinguishable species present in the country where the survey was conducted (Bashan et al. 2021). We made sure that the selected species are also common in cities and included the horse chestnut (*Aesculus hippocastanum*) as a widespread introduced urban species. The photographs were chosen to highlight distinct features clearly. Participants could first click on a picture to indicate that this species was familiar to them (familiarity, sensu Bashan et al. 2021). In the next step, they could write the name of the species (whether the colloquial name in German, English or the scientific name) in an open field (species identification skills, sensu Bashan et al. 2021). Here, correct answers were also given at the species, genus or family level, depending on the taxon (sensu Bashan et al. 2021). For instance, for horse chestnut, chestnut was counted as the correct answer, while ‘black bird with a bit of blue’ was deemed invalid for Eurasian magpie. Further, answers containing spelling mistakes, as long as the name was recognizable were also considered as correct. For instance, ‘berch’ was counted as a valid typo for Silver birch.

**Table 1:** Survey items and data used in the studies with adolescents, young and older adults

Section	Concept	Item	Data
1	Familiarity with species	*‘Check all the [plant, bird, butterfly] species that you know’.* Direct click on the shown photograph (Fig. 2)	Count data
1	Species identification skills	*‘What is the name of species [number of photograph]?’.* Open comment section below photographs (Fig. 2)	Count data of correct species names
2	Nature-relatedness	Subset of the NR6 scale (Nisbet and Zelenski 2013) with four items; sense of identification (self) and contact with nature (experience) to capture one’s general relationship with nature: *1.My ideal vacation spot would be a remote, wilderness area (experience)* *2. I always think about how my actions affect the environment (self)* *3. I take notice of wildlife wherever I am (experience)* *4. My relationship to nature is an important part of who I am (self)*	Ordinal (1 = do not agree at all to 5 = completely agree)
3	Pro-conservation behaviour intentions	Three items indicating likelihood to take following actions in the near future (Jacobs and Harms 2014): *1. Volunteer more for nature protection* *2. Motivate family and friends to volunteer more for animal and plant species* *3. Inform myself about nature protection, the environment, animals and plants*	Ordinal (1 = very unlikely to 5 = very likely)
4	Visit frequency	How often have you visited a public green space in the last four weeks (for example, park, playground, cemetery, area by the water, among others)?	Ordinal (1 = I have not visited a green space during the last four weeks/never, 2 = less than once a week, 3 = once a week, 4 = several times a week)
5	Age	Year of birth	Categorized in adolescents 15–17, young 18–29 and older adults > 30 years at the year of the survey for the analyses
	Gender	Male, female, nonbinary, genderfluid (only assessed in the adult populations)	
	Current place of living	Large city (> 100.000 inhabitants), medium city (> 20.000 inhabitants), small city (> 5000 inhabitants) and rural (> 5000 inhabitants)	Categorized into ‘urban’ (small to large city) and ‘rural’

In the second section, we asked participants about their nature-relatedness derived from the NR6 validated scale (Nisbet and Zelenski 2013). We used a subset of the NR6 scale with four items [vacation, action, wildlife, nature] focussing on the sense of identification (self) and contact with nature (experience) to capture one’s general relationship with nature (Table 1). In the third section, we asked participants about their intentions to get involved in pro-conservation behaviour [volunteer, motivate others, inform] adjusted from Jacobs and Harms (2014) and we defined pro-conservation behaviour intentions as the likelihood to take actions related to nature, animal or plant protection in the near future (Table 1). In the fourth section, we asked participants about the frequency they visited a public green space in the last four weeks (from never to several times a week) and in the last section, we asked participants for their year of birth, current place of living and gender; however, the latter was not included in the survey distributed in schools. We received feedback from the school supervisory authority recommending the omission of the gender question to ensure the anonymity of pupils. While the inclusion of gender among the pupils might have provided valuable insights, we respected this feedback to maintain participant comfort and focus on the key variables relevant to our study**.** Prior to sending out the final survey, we undertook a pilot study with participants (*n* = 20) from diverse socio-demographic backgrounds. Whenever necessary, we adjusted the items for clarity. Specifically, we focussed the original behaviour intention items from Jacobs and Harms (2014), on the protection of animals, plants and nature in general. Additionally, the NR6 scale was shortened to four items, as two items [‘My connection to nature and the environment is a part of my spirituality’, ‘I feel very connected to all living things and the earth’] were deemed too abstract by pupils.

### Survey population and distribution

We distributed the survey online to adolescents and adults using SoSci Survey^1^ as online was the only option during the COVID-19 pandemic. For the adolescent population, we contacted 237 schools in Berlin of which 10 responded and participated. Approval from the school supervisory authority in Berlin was obtained in accordance with the relevant legal regulation (SchulG 2004, article 65, subsection 2) and to ensure that the survey did not compromise educational activities. We selected pupils aged older than 15 years as participants of the survey and parental consent was not required for this group. Participants from schools had the option to access the online survey through a QR code (n = 8) with only two schools requesting a paper-format survey. Participants were allowed to complete the survey at their convenience. The survey was available in German from January 17, 2022 to March 03, 2022 within schools.

For the adult population aged 18 and above, the survey was distributed by various channels: we shared the survey in email distribution lists and Telegram groups that were not solely related to environmental interests (e.g. BIPoC (Black, Indigenous and People of Colour) but also environmental psychology mailing lists) which allowed us to capture responses from individuals with diverse backgrounds. We also asked to share the survey with other people, following a snowball technique (Bryman 2016). We further used online platforms, particularly Instagram, Facebook and Twitter. On Instagram and Twitter, the survey was shared through private profiles of one of the co-authors (MB). On Facebook, the survey was posted in groups dedicated to finding survey participants. Lastly, it was also distributed via the website SurveyCircle^2^. The survey for the adult population was available in both English and German from November 10, 2021, to January 10, 2022. While generalizing to the broader public is not possible with these convenience samples, they allow valuable opportunities for testing our theoretical framework.

### Statistical analyses

Statistical analyses were conducted using R version 4.2.3 (2023-03-15). Initially, we removed participants who skipped more than 10% of the answers from further analysis. Then, we assessed the internal validity of the nature-relatedness scale (NR6) subset and pro-conservation behaviour intentions with Cronbach’s alpha using the ‘psych’ package. The standardized Cronbach’s alpha for nature-relatedness was 0.73, 0.72 and 0.75 for adolescents, young adults and older adults, respectively, and for pro-conservation behaviour intentions, the values were 0.82 for adolescents, 0.81 for young adults and 0.70 for older adults. Consequently, we averaged the four items for NR6 and pro-conservation behaviour intentions into a composite scale. To quantify species identification skills and familiarity, we counted the number of species that participants correctly identified and marked as familiar, respectively.

For comparing visit frequency, species identification, familiarity with species, nature-relatedness and pro-conservation behaviour intentions between adolescents (15–17 years), young (18–29 years) and older adults (> 30 years), we applied a Kruskal–Wallis test followed by Dunn’s test with Bonferroni correction for multiple comparisons to determine statistical significance. Additionally, we conducted a Z-score analysis to identify and address potential outliers among pupils who were approached in schools and other participants from across Germany (verifying their German residency via their provided current postal code), minimizing the impact of extreme values in the samples. The percentage of outliers for nature-relatedness in the study population from across Germany was notably low at 0.32%, and for pro-conservation behaviour intentions, it was also minimal at 0.96%. All other values ranged between − 3 and + 3, indicating that no extreme outliers were found in either population.

To assess the correlation between concepts, we conducted a Spearman rank correlation (as the data were not normally distributed). While there are several cut-off points to describe the strength of the correlation between variables, we considered a coefficient < 0.39 a weak correlation, 0.40–0.69 a moderate and > 0.70 a strong correlation (Schober et al. 2018).

Following Otto and Pensini (2017), we adopted a unidirectional path model to test the theoretical framework (Fig. 1), examining the influence of visit frequencies on pro-conservation behaviour intentions through species identification skills (not familiarity) and nature-relatedness for each age category. Although there was a moderate relationship between species identification and familiarity in adolescents, and a stronger relationship in young and older adults (Table S1, Fig. S1), we focussed only on species identification in our path analysis, as this aligned more closely with our study's focus on how ‘accurate’ species knowledge relates to pro-conservation behaviour intentions. We also specified the path between species identification skills to nature-relatedness following the theoretical framework proposed by Clayton (2003) and Liefländer and Bogner (2014) that suggests that increasing environmental knowledge can foster a greater sense of connection to nature. Although a bidirectional relationship may exist, we focussed on a unidirectional model due to our interest in testing this specific direction of influence across age groups within the ‘cause’ and ‘consequence’ framework (Fig. 1).

In detail, we specified the path model for each population group using the ‘lavaan’ package (Rosseel 2012) and the ‘SEM’ function in R. The path analysis was set to eight parameters, using the maximum likelihood (ML) estimation and the NLMINB optimization method to ensure that parameters converged. The models tested direct and indirect effects among constructs: species identification was regressed on visit frequency, nature-relatedness was regressed on both species identification and visit frequency and pro-conservation behaviour intention was regressed on both nature-relatedness and species identification. This structure allowed us to test the effects of species identification on nature-relatedness and pro-conservation behaviour intentions, as well as the effect of nature-relatedness on pro-conservation behaviour intentions. Additionally, it tested the roles of nature-relatedness and species identification as path variables between visit frequency and pro-conservation behaviour intentions. The fit of each model was evaluated using Chi-square test statistics, comparative fit index (CFI), Tucker–Lewis index (TLI) and the root mean square error of approximation (RMSEA). We considered a CFI ≥ 0.95, a TLI ≥ 0.95 and an RMSEA < 0.06, as indicators of acceptable model fit based on Hu and Bentler (1999)

## Results

### Survey population

In total, we received 600 responses with n = 252 participants between 15 and 17 years old (mean = 15.97, median = 16, SD = 0.74; *hereafter* adolescents), n = 215 participants between 18 and 29 years old (mean = 23.6, median = 24, SD = 3.65; *hereafter* young adults) and *n* = 133 participants between 30 and 76 years old (mean = 40.54, median = 36, SD = 11.35; *hereafter* older adults). Most participants visited green spaces several times a week (*n* = 247), followed by once a week (*n* = 171), less than once a week (*n* = 139) and rarely or never (*n* = 41). All adolescents had an urban background (100%) at the time of the survey. Similarly, most young and older adults had an urban background (95% and 89%, respectively). Among participants surveyed outside of schools, females were the largest group (*n* = 209), followed by males (*n* = 82). A smaller number identified as genderfluid (*n* = 4) and nonbinary (*n* = 3). Although gender was not assessed for pupils, all participating schools provided co-educational (i.e. mixed-gender) education.

### Differences among age categories

**Fig. 3:**
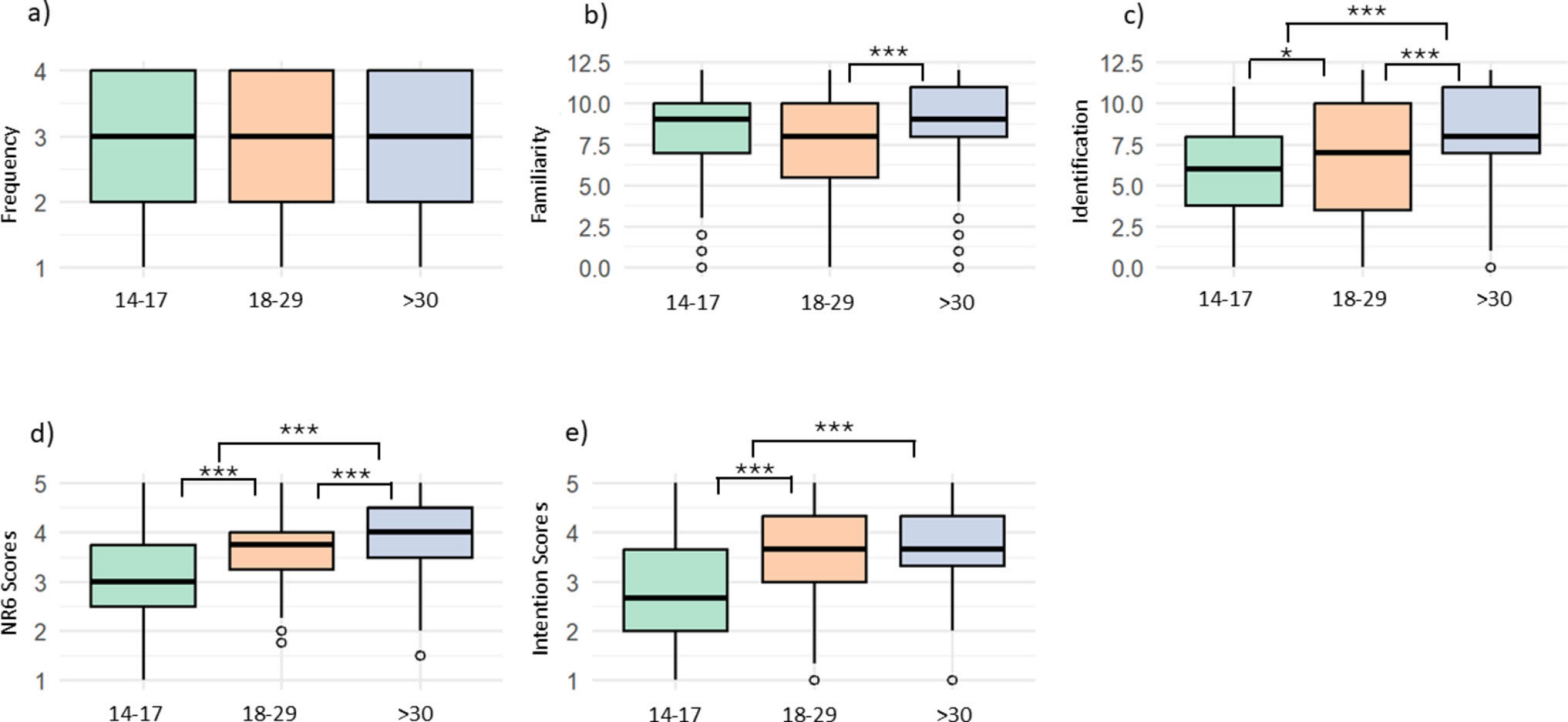
Differences between adolescents (14–17 years), young (18–20 years) and older adults (> 30 years): **a** visit frequency to green spaces (scale 1–4), **b** familiarity with species (overall, with the 12 species of plants, butterflies and birds combined), **c** species identification skills (overall, with the 12 species of plants, butterflies and birds combined), **d** nature-relatedness (scale 1–5) and **e** pro-conservation behaviour intentions (scale 1–5) (see Table 2 for values and Table 1 for scales). Significant differences based on Dunn’s test with Bonferroni correction are marked as *** *p* < 0.001 and * *p* < 0.05; if not marked, differences are not statistically significant

Overall, we observed significant differences in all assessed concepts, except for visit frequency (Table 2, Fig. 3). We found the most significant differences in species identification skills and nature-relatedness among the three age categories. Older adults, scoring an average of 8.09 out of 12, had significantly better species identification skills than young adults (scoring 6.38) and adolescents (scoring 5.87) (Kruskal–Wallis χ^2^ = 43.32, *p* < 0.001). They also had a significantly higher nature-relatedness score, averaging 3.98 out of 5, compared to young adults (scoring 3.61) and adolescents (scoring 3.09) (Kruskal–Wallis χ^2^ = 113.62, *p* < 0.001). Among the younger age categories, young adults scored significantly higher than adolescents in both species identification skills and nature-relatedness (*p* < 0.05 for identification skills and *p* < 0.001 for nature-relatedness, according to Dunn’s test with Bonferroni correction).

**Table 2:** Age category differences in visit frequency, familiarity with species, species identification skills, nature-relatedness and pro-conservation behaviour intentions (see Fig. 3). The table presents average scores, along with medians and standard deviations. Significant differences were determined using the Kruskal–Wallis test and Dunn’s post hoc test with Bonferroni correction

	Age categories	n	Mean	SD	Median	χ^2^	P	Significant differences
Visit frequency	Adolescents	252	3.08	0.95	3.0	2.95	0.23	
	Young adults	215	2.95	0.99	3.0			
	Older adults	133	3.14	0.90	3.0			
Familiarity	Adolescents	252	8.20	2.87	9.00	14.64	< 0.001	Older adults–young adults
	Young adults	215	7.46	3.67	8.00			
	Older adults	133	8.79	2.82	9.00			
Species identification	Adolescents	252	5.87	2.92	6.00	43.32	< 0.001	Older adults–young adult older adults–adolescents Young adults–adolescents
	Young adults	215	6.38	3.68	7.00			
	Older adults	133	8.09	3.15	8.00			
Nature-relatedness	Adolescents	252	3.09	0.77	3.00	113.62	< 0.001	Older adults–young adult older adults–adolescents Young adults–adolescents
	Young adults	215	3.61	0.72	3.75			
	Older adults	133	3.98	0.70	4.00			
Pro-conservation behaviour intention	Adolescents	252	2.82	0.99	2.67	98.38	< 0.001	Older adults–adolescents Young adults–adolescents
	Young adults	215	3.55	0.94	3.67			
	Older adults	133	3.76	0.77	3.67

As for pro-conservation behaviour intentions, older (scoring 3.76 out of 5) and young adults (scoring 3.55) scored significantly higher than adolescents (scoring 2.82) (Kruskal–Wallis χ^2^ = 98.38, *p* < 0.001; Dunn’s test with Bonferroni correction). However, there were no significant differences in pro-conservation behaviour intentions between young and older adults (*p* > 0.05; Dunn’s test with Bonferroni correction).

Regarding familiarity with species, older adults reported significantly greater familiarity (scoring 8.79 out of 12) compared to young adults (scoring 7.46) (Kruskal–Wallis χ^2^ = 14.64, *p* < 0.001; Dunn’s test with Bonferroni correction). But there were no significant differences of both age categories compared to adolescents (scoring 8.20) (*p* > 0.05; Dunn’s test with Bonferroni correction). Lastly, we found no significant differences in visit frequency among the age categories (Kruskal–Wallis χ^2^ = 2.95, df = 2, *p* = 0.228).

### Identification of and familiarity with butterfly, bird and plant species

Overall, adolescents, young and older adults showed the highest familiarity and identification skills with plants, followed by birds and butterflies (Table 3). The species most frequently correctly named across all three taxonomic groups were stinging nettle (86%), House sparrow (67.3%) and brimstone (58.2%). The species least frequently correctly named across all three taxonomic groups were the horse chestnut (52.8%), Eurasian magpie (41.5%) and small tortoiseshell (10.8%). None of the 12 species was correctly identified by all participants.

**Table 3:** Percentage of correct species identification and self-determined familiarity among participants. Species of plants, birds and butterflies are sorted after decreasing percentage of familiarity. Significant differences among age categories were derived using the Kruskal–Wallis test and Dunn’s post hoc test with Bonferroni correction

Group	Species	Age category	Familiarity (%)		χ^2^	*p*	Significant differences	Species identification (%)		χ^2^	*p*	Significant differences
Plants	Blackberry (*Rubus fruticosus* (coll.))	Adolescents	92.7	92.1	0.48	0.79	–	76.3	72.6	5.65	0.06	–
		Young adults		92.6					76.3			
		Older adults		94.0					83.5			
	Stinging nettle (*Urtica dioica*)	Adolescents	88.3	90.9	3.17	0.21	–	86.0	88.9	4.96	0.08	–
		Young adults		85.6					81.9			
		Older adults		88.0					87.2			
	Silver birch (*Betula pendula*)	Adolescents	85.5	87.7	7.06	0.029	Older adults–young adults Young adults–adolescents	77.8	77.0	13.32	0.001	Older adults–adolescents Young adults–adolescents
		Young adults		80.5					72.1			
		Older adults		89.5					88.7			
	Horse chestnut (*Aesculus hippocastanum*)	Adolescents	73.2	68.3	14.18	< 0.001	Older adults–young adults Young adults–adolescents	52.8	38.9	45.26	< 0.001	Older adults–adolescents Young adults–adolescents
		Young adults		71.2					55.8			
		Older adults		85.7					74.4			
Birds	Common blackbird (*Turdus merula*)	Adolescents	80.2	82.1	6.30	< 0.05	Older adults–young adults	67.0	61.1	13.71	0.001	Older adults–adolescents Young adults–adolescents
		Young adults		74.9			Older adults–adolescents		66.0			
		Older adults		85.0			Young adults–adolescents		79.7			
	House sparrow (*Passer domesticus*)	Adolescents	80.7	89.7	34.58	< 0.001	Older adults–young adults	67.3	73.8	18.61	< 0.001	Older adults–adolescents Young adults–adolescents
		Young adults		68.4			Older adults–adolescents		56.3			
		Older adults		83.5			Young adults–adolescents		72.9			
	European robin (*Erithacus rubecula*)	Adolescents	71.7	63.5	21.27	< 0.001	Older adults–young adults	64.7	52.4	39.31	< 0.001	Older adults–adolescents Young adults–adolescents
		Young adults		72.6			Older adults–adolescents		67.0			
		Older adults		85.7			Young adults–adolescents		84.2			
	Eurasian magpie (*Pica pica*)	Adolescents	51.7	45.2	21.39	< 0.001	Older adults–young adults Older adults–adolescents Young adults–adolescents	41.5	29.4	36.28	< 0.001	Older adults–adolescents Young adults–adolescents
		Young adults		48.4					43.7			
		Older adults		69.2					60.9			
Butterflies	Brimstone (*Gonepteryx rhamni*)	Adolescents	62.0	67.5	10.44	0.005	Older adults–adolescents Young adults–adolescents	58.2	58.3	6.96	< 0.001	Older adults–adolescents Young adults–adolescents
		Young adults		53.5					52.6			
		Older adults		65.4					66.9			
	Small white (*Pieris rapae*)	Adolescents	30.2	30.6	6.09	0.048	Older adults–young adults Young adults–adolescents	17.7	7.9	42.63	< 0.001	Older adults–adolescents Young adults–adolescents
		Young adults		25.1					18.6			
		Older adults		37.6					34.6			
	Peacock (*Aglais io*)	Adolescents	27.5	27.5	225.11	< 0.001	Older adults–adolescents Young adults–adolescents	34.5	24.2 34.4 54.1	6.96	0.03	Older adults–adolescents Young adults–adolescents
		Young adults		7.0					34.4			
		Older adults		0					54.1			
	Small tortoiseshell (*Aglais urticae*)	Adolescents	19.7	42.9	148.78	< 0.001	Older adults–adolescents Young adults–adolescents	10.8	2.8	35.01	< 0.001	Older adults–adolescents Young adults–adolescents
		Young adults		4.7					13.5			
		Older adults		0					21.8			

All participants correctly identified more than 50% of the plant and bird species except for the horse chestnut which was only identified by 38.9% correctly among adolescents. The Eurasian magpie was only identified by 43.7% correctly among young adults and 29.4% among adolescents. Butterflies presented the greatest challenge, with only the brimstone butterfly being correctly identified above 50% among all age categories. The small tortoiseshell was only correctly identified between 21.8% (older adults), 13.5% (young adults) and 2.8% (adolescents).

Overall, when species identification was high, familiarity was also and vice versa. However, within the butterfly category, we observed the largest discrepancies between familiarity and species identification among age groups. While adolescents reported higher levels of familiarity with butterfly species compared to young and old adults, they were less successful in correctly identifying these species; particularly in the case of the small tortoiseshell.

### Relationship between concepts

In all three age categories, nature-relatedness was significantly correlated with pro-conservation behaviour intentions, showing moderate correlations in adolescents (rho = 0.51), young adults (rho = 0.48) and older adults (rho = 0.47) (Table S1, Fig. S1). Species identification skills and familiarity were also significantly correlated with each other, showing a moderate correlation in adolescents (rho = 0.57) and strong correlations in young adults (rho = 0.89) and older adults (rho = 0.79). Additionally, species identification skills and familiarity were positively associated with both nature-relatedness and pro-conservation behaviour intentions in adolescents and young adults. These correlations were weak in adolescents (rho = 0.28 and rho = 0.26 for nature-relatedness, and rho = 0.25 and rho = 0.19 for pro-conservation behaviour intentions) and moderate to weak in young adults (rho = 0.41 and rho = 0.33 for nature-relatedness, and rho = 0.27 and rho = 0.25 for pro-conservation behaviour intentions), which likely explains why they did not show significant effects in the path analyses. While similar patterns were found for older adults, this was not significant in the case of pro-conservation behaviour intentions (rho = 0.16 for both pro-conservation behaviour identification and familiarity). The differences among age categories were that only young adults showed significant positive correlations between frequency and familiarity (rho = 0.19), frequency and species identification (rho = 0.28) and familiarity and pro-conservation behaviour intentions (rho = 0.25).

### Pathways between concepts

**Fig. 4:**
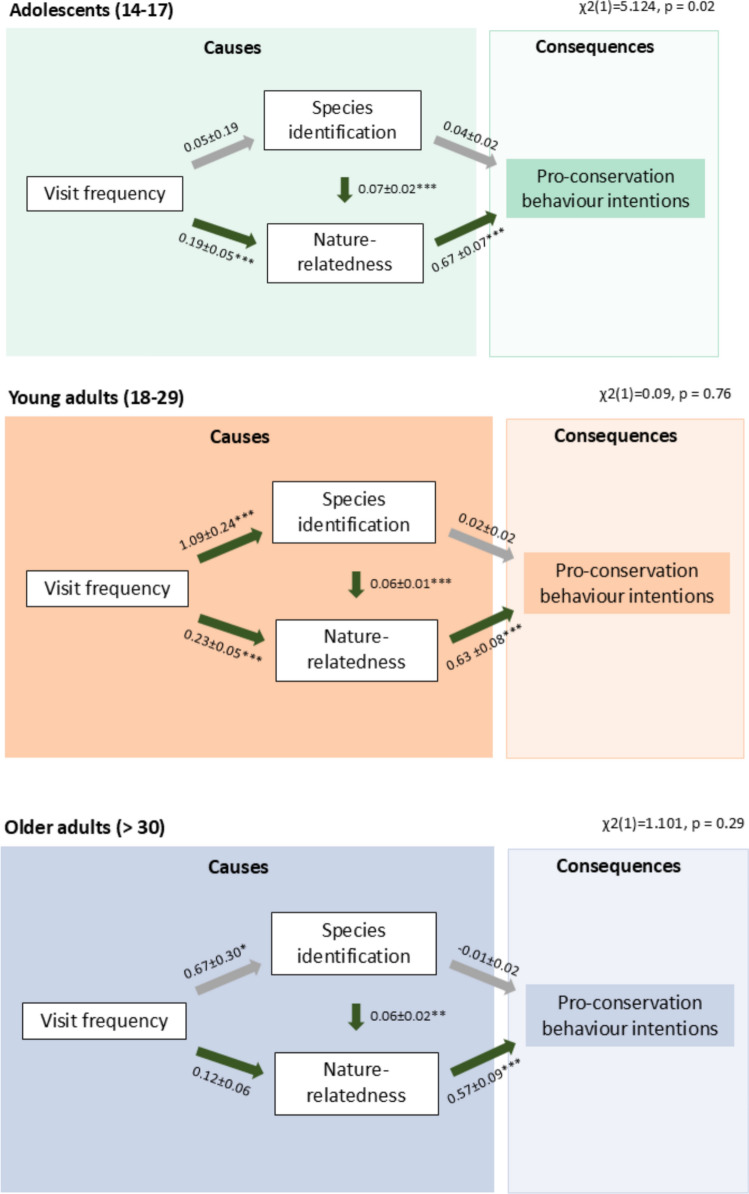
Path analyses for adolescents (top), young (middle) and older adults (bottom) showing the relationship between visit frequencies, identification skills, nature-relatedness and pro-conservation behaviour intentions. Green arrows indicate positive significant relationships with * *p* < 0.05, ** *p* < 0.001 and *** *p* < 0.001 levels, whereas grey arrows indicate no significant relationship. Chi-square test statistics are shown at the top right

In all three age categories, the path model showed a significant pathway between species identification and nature-relatedness as well as between nature-relatedness and pro-conservation behaviour intentions (Fig. 4, Table S2). The models for the young and older adult populations demonstrated an excellent fit to the data. For the young adults, the Chi-square test statistic was nonsignificant (χ^2^(1) = 0.092, *p* = 0.762), with CFI = 1.00, TLI = 1.04 and RMSEA < 0.001 (95% CI 0.00–0.124). Similarly, the older adult model also showed a nonsignificant Chi-square test statistic (χ^2^(1) = 1.101, *p* = 0.294), with CFI = 0.998, TLI = 0.909 and RMSEA = 0.028 (95% CI 0.00–0.235).

In contrast, the adolescent population model indicated a poorer fit to the data. The Chi-square test statistic was significant (χ^2^(1) = 5.12, *p* = 0.02), suggesting a substantial discrepancy between the model and the observed data. Although the CFI was 0.967, the TLI was 0.803 and the RMSEA was 0.128 (95% CI 0.038–0.247), confirming an unacceptable fit of the model to the adolescent population data.

The young adult population (*n* = 215) model showed a strong and significant positive relationship between visit frequency and species identification (*β* = 1.088, *p* < 0.001). Visit frequency had a significant positive effect on nature-relatedness (*β* = 0.229, *p* < 0.001) and species identification was a significant predictor of nature-relatedness (*β* = 0.058, *p* < 0.001) but not of pro-conservation behaviour intentions (*β* = 0.021, *p* = 0.21). Nature-relatedness was a strong and significant predictor of pro-conservation behaviour intentions (*β* = 0.634, *p* < 0.001).

Similar to the young adult population, the older adult population (*n* = 133) model demonstrated a significant positive relationship between visit frequency and species identification (*β* = 0.670, *p* = 0.025) and species identification remained a strong predictor of nature-relatedness (*β* = 0.063, *p* = 0.001) but not of pro-conservation behaviour intentions (*β* = − 0.005, *p* = 0.777). Nature-relatedness remained a significant predictor of pro-conservation behaviour intentions in the adult populations (*β* = 0.568, *p* < 0.001). In contrast to young adults, visit frequency was in the older adult population only a marginal but not a significant predictor of nature-relatedness (*β* = 0.119, *p* = 0.063).

Considering the adolescent population (*n* = 252) model, visit frequency showed similar as in the young adult population a positive and significant effect on nature-relatedness (*β* = 0.19, *p* < 0.001). Similar to the young and older adult population, nature-relatedness was a significant predictor of pro-conservation behaviour intentions (*β* = 0.67, *p* < 0.001) and species identification significantly predicted nature-relatedness (*β* = 0.07, *p* < 0.001). In contrast to young and older adults, visit frequency was no significant predictor of species identification (*β* = 0.05, *p* = 0.79). However, path coefficients in the adolescent model should be interpreted with caution, as the model showed a poor fit to the data.

## Discussion

Understanding the drivers of pro-conservation engagement in current and future generations is crucial for counteracting the global biodiversity crisis. Previous research has uncovered various relationships between opportunities to experience nature, nature exposure, people’s orientation towards nature and pro-conservation intentions (Lin et al. 2014; Collado et al. 2015; Hughes et al. 2018; Whitburn et al. 2020). However, there is a limited understanding of how these concepts differ across different age categories and whether their interrelationships change with age. This gap was addressed by our study, which explored the levels of green areas visit frequencies, species identification skills and familiarity with species, nature-relatedness and pro-conservation behaviour intentions in adolescent, young and older adult populations in Germany. In the second step, we used path analyses to assess the relationships among concepts (excluding familiarity). Our study revealed significant differences in species identification, nature-relatedness and pro-conservation behaviour intentions across age categories. Younger participants scored lower in species identification (supporting the generational amnesia phenomenon), nature-relatedness (supporting the extinction of experience phenomenon) and pro-conservation behaviour intentions compared to older participants. Interestingly, visit frequency (part of the extinction of experience phenomenon) did not differ among age categories. Lastly, we found consistently across all age groups that nature-relatedness had a strong positive effect on pro-conservation intentions and that species identification was a significant positive predictor of nature-relatedness. Consequently, species identification indirectly influenced pro-conservation intentions through its effect on nature-relatedness.

### Approaches towards understanding causes and consequences of extinction of experience

In their framework on the extinction of experience, Soga and Gaston (2016) identified opportunity and orientation as the key causes of this phenomenon, with crucial consequences on people’s behaviour. We did not assess opportunities in our study, but visit frequency to public green sites since nature exposure is an important component of both opportunity and orientation (Lin et al. 2014; Soga and Gaston 2016). We decided for this approach as simply having green spaces nearby does not guarantee they are used as barriers exist in different population groups to visiting accessible green areas (De Haas et al. 2021; Waite et al. 2023; Langhans et al. 2023). Accordingly, an Australian study revealed that not the distance to natural space, but the activities performed in such spaces were related to people’s support for conservation (Dean et al. 2019).

Orientation has been broadly characterized as the motivation to engage with nature (Gaston and Soga 2020). As indicators of orientation, we used species knowledge, specifically, identification skills and nature-relatedness as a measure of a person’s relationship with nature. We chose this approach based on the assumption that knowledge of species can motivate more meaningful engagement with nature and foster pro-conservation actions (van Heezik et al. 2020). Furthermore, a stronger relationship with nature has been well established to influence pro-conservation actions (Mayer et al. 2009; Whitburn et al. 2019). Hence, we consider both species knowledge and nature-relatedness as strong motivators for engaging with nature, with the potential to foster conservation-oriented actions.

### Similar visit frequency across age categories

Contrary to the expectations from the phenomenon of extinction of experience, which suggests that contact with nature is decreasing (Soga and Gaston 2016), our study found that adolescents, young and older adults reported similar frequencies of visiting green areas. Previous studies showed mixed results in this context. Novotný et al. (2021) did not find a decrease in outdoor activities among children and, similarly, Oh et al. (2020) did not observe a decrease in green space visits in Singapore between 1996 and 2018. Instead, the latter study found a tendency for the study population in 2018 to use green spaces more frequently and for longer durations than in 1996. Conversely, other studies have reported a decline in nature exposure of younger generations (Soga and Gaston 2016; Larson et al. 2019). The similar visit frequency across age groups in our study suggests equal opportunities to interact with nature in green spaces, though the approaches to these interactions are likely to differ. The lower levels of identification skills, nature-relatedness and pro-conservation behaviour intentions in younger participants compared to older ones indicate that park visits do not always strengthen the orientation towards nature, nor do they necessarily increase intentions to engage in pro-conservation actions.

### Decreasing identification skills from older to younger participants

Our findings support the generational amnesia hypothesis as younger participants showed lower identification skills than older ones. This aligns with another German study where bird recognition by adults increased with age (Enzensberger et al. 2022) and a study in Israel where familiarity and species identification skills for plants, birds and butterflies similarly significantly increased with age (Bashan et al. 2021). This trend can also be found in time comparison by Gerl et al. (2021) who compared vertebrate knowledge among 11–13-year-old students in 2018 to the same age category in 2006, revealing a 15% decrease within a bit more than a decade.

Our study contributes to this body of knowledge by applying the same methodology for assessing identification skills of common plant, bird and butterfly species as Bashan et al. (2021) in Israel and hence, enables us to draw careful comparisons. First, in our but also Bashan et al.’s (2021) study, there was a consistent trend of decreasing species identification from plants to birds to butterflies, indicating a general pattern across generations but in different cultural contexts. Second, across all age categories, the overall species identification in our study was higher than in Bashan et al.’s (2021) study. Participants in the latter study identified an average of one-third of the presented species correctly (3.83 out of 12), while this proportion was almost half (5.75 out of 12 species) in our study. However, neither our study nor that of Bashan et al. (2021) can make generalizations about the German or Israeli populations, respectively. Two other studies that used slightly different methods to assess species knowledge found, for example, that in the UK, participants identified birds, particularly blue tits, better than plants (Dallimer et al. 2012). And, a comparative study evaluating species knowledge across three countries (the UK, France and Israel) found that participants in the UK scored highest in species identification skills, followed by France, with Israel scoring the lowest (Colléony et al. 2019).

### Decreasing nature-relatedness from older to younger participants

As hypothesized by the extinction of experience theory, we found that younger participants demonstrated lower nature-relatedness than older ones, suggesting a diminishing orientation towards nature in younger individuals. While our study was cross-sectional in a certain period, the time comparison study by Oh et al. (2020) observed a similar trend in Singapore, where nature connections among residents declined between 1996 and 2018. While the extinction of experience theory considers declining opportunities as one driver of decreasing nature-relatedness (Soga and Gaston 2016), age categories in our study differed in the level of nature-relatedness despite similar visit frequencies to green areas. This confirms that simply spending time in nature does not automatically enhance nature connection (Richardson et al. 2022). Dog walking, for example, is regularly associated with exposure to green areas, but it did not correlate with dog walkers’ knowledge about nature, their environmental attitudes (Colléony et al. 2019) or their valuation of biodiversity (Fischer and Kowarik 2020).

Given the importance of the social and cultural context in interactions with nature (Clayton et al. 2017), the reasons to visit green areas for specific activities may differ across age categories. In a Swedish city, older people have been found to engage more in nature-related activities than younger people (Sang et al. 2016). Park uses differed across age categories in a pan-European study, with younger people being less engaged in nature-related activities in Berlin than older ones (Fischer et al. 2018). Younger people may engage in different activities, for example, with their mobile devices and social media instead of fully experiencing their natural surroundings (Clayton et al. 2017; Toh et al. 2019). Such shifts in activity may explain the observed decline in identification skills and nature-relatedness within our study population, despite similar frequencies of nature visits.

### Decreasing pro-conservation behaviour intentions from adults to adolescents

Adolescents in our study showed significantly lower scores of pro-conservation intentions than younger and older adults. Given the similar patterns observed for nature-relatedness, we consider this as empirical support for the concern that a disconnection from nature of younger generations leads to lower care for nature (Chawla 2020; Maleknia et al. 2024). While we did not test this, we acknowledge again the bidirectionality of our framework (i.e. more care for nature can lead into spending more time in nature). A recent study on the intentions of younger generations to participate in activities to conserve sacred trees also showed lower intentions and behaviours towards conservation than older generations (Maleknia et al. 2024). The authors claimed more targeted conservation initiatives to address the specific concerns and motivations of younger generations.

We found differences in pro-conservation behaviour intentions only between adolescents and both younger and older adults. Our analyses used age-based categories aligned with biological age that we use to reflect on the environmental concerns of our study population. Our ‘adolescents’ (15–17 years) population aligns with the so-called ‘Generation Z’ (born 1997–2012). This generation is known for its climate change and eco-anxiety, which leads to worry, distress and actions aimed at addressing these crises (Tsevreni et al. 2023). Despite this sensitivity to environmental concerns, the pro-environmental behaviour intentions in our youngest age category were lower than in older participants. Our items focussed broadly on animal, plant and nature protection, but more targeted items such as those related to biodiversity conservation behaviours like advocacy, sustainable consumption, donations, lifestyle choices, social engagement or stewardship might have been more appropriate (Selinske et al 2020). Scales specifically designed to measure conservation behaviours also exist (Barbett et al. 2020). However, beyond the possible limitations of our scale, another obvious possibility is that adolescents feel disillusioned about engaging in pro-conservation behaviours and were hence, lower in their scores.

### Pathways between visit frequency and pro-conservation intentions

Previous studies (Gosling and Williams 2010; Geng et al. 2015; Barrera-Hernández et al. 2020) have already shown that nature-relatedness is linked to pro-conservation behaviour intentions. Our study adds the insight that the pathway from nature-relatedness to pro-conservation behaviour intentions remained stable across all three age categories (although with a low model fit observed in the adolescent population) In addition, we found that species identification skills had an indirect impact on pro-conservation behaviour intentions via nature-relatedness, consistently across age categories. This finding highlights the importance of both components of our orientation framework: nature-relatedness which fosters pro-environmental behaviours (Mayer et al. 2009; Whitburn et al. 2019), but also species knowledge which reinforces this connection with nature (Clayton 2003; Liefländer and Bogner 2014). Although species knowledge did not directly influence pro-conservation behaviour intentions, it did have an indirect influence which indicates its complementary role besides nature-relatedness. Additionally, given the critical role of species knowledge, it is concerning to note a lack of species knowledge also among educators (Frobel and Schlumprecht 2016). Addressing this gap should be a focus to address the decreased identification skills among future generations. Experiencing nature is widely considered essential for building connections to nature (Bratman et al. 2015; Freeman et al. 2021). However, our results only significantly support this link between visit frequency and nature-relatedness clearly for the young adult population (as it was not significant for the older adult population). While correlation does not imply causality, we interpret the missing strong evidence between visit frequency and nature-relatedness again in that the mere opportunity for contact with nature is not sufficient to deeply connect with it, as discussed above about dog walking. The key question seems to be how individuals choose to utilize the opportunities to interact with nature that visiting green areas offers (Freeman, Harris and Loynes, 2021). Some interactions may lead to stronger nature-relatedness than others. For instance, Colléony et al. (2020b)showed in their experimental study that when they provided participants ‘cues to care for nature’ such as smelling flowers and observing wildlife this would lead to a stronger nature-relatedness. Such interactions have been quantified for parks in Berlin, showing that only 12% of observed park visitors engaged in species-related activities like foraging for wild plants (Palliwoda et al. 2017). The latter activity has been shown to enhance nature-relatedness (Schunko and Brandner 2022). Further, the sociocultural context is crucial to consider when it comes to interactions with nature (Clayton et al. 2017).

Within the young adult population, visit frequency positively influenced not only nature-relatedness but also species knowledge, a trend also observed in the older adults. This suggests that natural settings can serve as valuable learning environments. One result that contrasts with previous studies (Otto and Pensini 2017; Enzensberger et al. 2022; Härtel et al. 2023) is that we did not find evidence for a direct link between species knowledge and pro-conservation behaviour intentions. However, the indirect link (via nature-relatedness) supports the idea that knowledge about nature alone does not enhance pro-conservation intentions and reinforces the necessity of a deeper connection with nature (Kaplan et al. 1998; Miller 2005). While earlier studies have already shown that knowledge is essential for biodiversity conservation (Hooykaas et al. 2019; Skarstein and Skarstein 2020) and for fostering a connection with nature (Clayton 2003; Liefländer and Bogner 2014), our study contributes the new insight that the link between species knowledge and nature-relatedness remains stable across all age categories.

### Enhancing opportunities to connect with nature

Given the strong relationship between nature-relatedness and pro-conservation behaviour in our study, opportunities should be enhanced in urban environments in which younger as well as older residents can experience nature and build a relationship with it. While our primary focus was on the role of nature-relatedness in fostering pro-conservation behaviour intentions, further positive effects are associated with a deeper relationship with nature, namely well-being, health, happiness, and a satisfying and meaningful life (Nisbet and Zelenski 2013; Zylstra et al. 2014).

An example for enhancing opportunities to connect with nature is urban foraging, often an underestimated activity of collecting wild plants, where knowledge about the species is combined with their use for salads, teas and other purposes (Shackleton et al. 2017; Fischer and Kowarik 2020). This can also be integrated into school activities (Fischer et al. 2019). Learning opportunities beyond schools should also provide opportunities in which younger generations could also learn from older generations (Ardoin et al. 2020). Establishing signs in nature inspiring people to interact with natural elements can also foster stronger nature-relatedness (Colléony et al. 2020a, b). Lastly, as Papworth et al. (2009, p. 89) clearly pointed out in their work on generational amnesia, environmental education should ‘*ensure that different generations communicate about changes in their environment and establish an accurate narrative’*.

### Limitations

There are several limitations in our study. One consideration should be given to the recruitment of our participants. Half of our participants (n = 298, 15–19 years) were recruited among pupils in Berlin, a major city in Germany, and further participants (n = 302, > 18 years) with a predominantly prevailing urban background throughout Germany. However, all participants received the same survey within a period of five months under as similar conditions as possible, i.e. online, and partially in paper format. Further, the Z-scores indicated a uniform distribution of data across both populations without significant outliers. The recruitment outside schools was undertaken through mailing lists and social media platforms, which may have resulted in a younger demographic than would have been obtained through other distribution methods, such as local news magazines. This convenience and snowball sampling approach likely favoured individuals who are more active on digital platforms. Consequently, we acknowledge that the sample does not fully represent the broader adult population in Germany; e.g. we had a bias towards younger females. Future research could benefit from a more diverse recruitment strategy to capture a wider age range and better reflect the general adult population. We would expect an even stronger difference in identification and nature-relatedness in this case.

The low model fit observed in adolescents is particularly interesting in this context. While this framework, based on Otto and Pensini (2017), performed well with children in their study and with both younger and older adults in our study, it raises questions about the framework’s applicability across age groups. Specifically, it prompts consideration of whether other factors, which we did not account for, should be considered when examining the adolescent population. We further acknowledge that our measures of pro-conservation behaviour intentions were broadly selected rather than being more specifically related to biodiversity conservation behaviours (Selinske et al. 2020) which could have provided clearer patterns in our results. Additionally, factors that may have influenced responses regarding the frequency of visiting green spaces (i.e. nature exposure) include the timing of the survey during the COVID-19 pandemic. This period resulted in global disparities in urban green space use, with differences between age groups with older people experiencing greater obstacles in accessing urban green spaces than younger ones (Kleinschroth et al. 2024). We also did not differentiate between the quality of nature experience, i.e. which activities participants performed in the visited green spaces which could have been informative to our results. Other limitations include the social desirability bias, i.e. that participants responded in a way as they thought it would have been expected from them. However, the extent to which this potential bias influences results still remains questionable (Otto and Pensini 2017).

## Conclusions

Our study highlights the direct role of nature-relatedness and indirect role of species identification skills for pro-conservation behaviour intentions in adolescents, young adults and older adults. We confirmed that identification skills, nature-relatedness and pro-conservation behaviour intentions decreased from older to younger participants, despite similar visit frequencies to green spaces. This has implications for conservation education, policy development and research.

While the opportunity to experience nature is important, this may not be sufficient to drive pro-conservation behaviour alone. A deeper relationship with nature, which is what nature-relatedness represents, and knowledge about nature are both important in motivating people; irrespective of age, to engage in conservation efforts. Urban green (but also blue) spaces provide chances to learn and meaningfully interact with nature. Here, policy makers might consider strategies that combine access to natural areas with opportunities to connect with nature (e.g. ‘cues to care for nature’, Colléony et al. 2020a, b) and environmental education (Ardoin et al. 2020). The identified hierarchy of species identification and familiarity decreasing from plants, birds to butterflies should encourage efforts to include invertebrates as an ecological highly important but less known group of taxa in educational programmes.

Promising future avenues to explore are the impacts of different nature experiences (e.g. also across seasons) on the nature-relatedness and species knowledge within various sociocultural groups (including different age groups) but also investing additional moderators and mediators in the relationship between nature exposure and conservation behaviour (Soga and Gaston 2024). For instance, while we focussed on species knowledge and nature-relatedness as measures of orientation, other factors such as sense of place or perceived behaviour control (the degree to which individuals feel capable of making a difference) could be further interesting concepts to study the concept of orientation, i.e. the motivation to engage with nature. These could inform age-specific strategies that facilitate meaningful interactions with nature that also inspire visitors in their support for conservation efforts.

## Supplementary Information

Below is the link to the electronic supplementary material.

Supplementary file1.

## Data Availability

The data will be available in Dryad once published.

## Footnotes

1 www.soscisurvey.de.

2 www.surveycircle.de.
