# Supplementary material for: From nature experience to pro-conservation action: How generational amnesia and declining nature-relatedness shape behaviour intentions of adolescents and adults
Source: Ambio. 2025 Mar 6;54(7):1165–84. doi: 10.1007/s13280-025-02135-7 (PMC12133659; doi:10.1007/s13280-025-02135-7)
Supplement: Supplementary file 1 — Supplementary file1. [file 13280_2025_2135_MOESM1_ESM.pdf]

Supplementary information

Title: From nature experience to pro-conservation action: how generational amnesia and declining nature-relatedness shape behaviour intentions of adolescents and adults

This supplementary information has not been peer reviewed

Tables

Table S1: Spearman correlation coefficients (rho) between frequency of visiting green spaces (Frequency), nature-relatedness (Nat.relat.), identification skills (Identification), familiarity with species (Familiarity) and pro-conservation behaviour intentions (Intentions) for adolescents, young and older adults. The significance of the correlations is indicated with asterisks: *** p < 0.001, ** p < 0.01, * p< 0.05 .

Adolescents	Frequency	Nat.relat.	Familiarity	Identification	Intentions
Frequency	1.00	**0.25*** **	0.03	0.01	0.00
Nat.relat.	**0.25*** **	1.00	**0.26*** **	**0.28*** **	**0.51*** **
Familiarity	0.03	**0.26*** **	1.00	**0.57*** **	**0.19** **
Identification	0.01	**0.28*** **	**0.57*** **	1.00	**0.25*** **
Intentions	0.00	**0.51*** **	0.19	**0.25*** **	1.00
Young adults					
Frequency	1.00	**0.41*** **	**0.19* **	**0.28*** **	**0.25*** **
Nat.relat.	**0.41*** **	1.00	**0.33*** **	**0.41*** **	**0.48*** **
Familiarity	**0.19* **	**0.33*** **	1.00	**0.89*** **	**0.25*** **
Identification	**0.28*** **	**0.41*** **	**0.89*** **	1.00	**0.27*** **
Intentions	**0.25*** **	**0.48*** **	**0.25*** **	**0.27*** **	1.00
Older adults					
Frequency	1.00	**0.21* **	0.09	0.14	0.05
Nat.relat.	**0.21* **	1.00	**0.33*** **	**0.34*** **	**0.47*** **
Familiarity	0.09	**0.33*** **	1.00	**0.79*** **	0.16
Identification	0.14	**0.34*** **	**0.79*** **	1.00	0.16
Intentions	0.05	**0.47*** **	0.16	0.16	1.00

Table S2: Results of the path analysis with the estimated path coefficients (estimate), standard errors (S.E.), z-values (z-value), and p-values (p-value) for adolescents, young adults, and older adults.

	**Estimate**	**S.E.**	**z-value**	**p-value**
Adolescents				
Identification				
Frequency	0.052	0.194	0.265	0.791
NVmean				
Frequency	0.189	0.048	3.916	0.000
Identification	0.068	0.016	4.356	0.000
BImean				
NVmean	0.671	0.070	9.633	0.000
Identification	0.035	0.018	1.916	0.055
Young adults				
Identification				
Frequency	1.088	0.242	4.501	0.000
NVmean				
Frequency	0.229	0.046	5.010	0.000
Identification	0.058	0.012	4.612	0.000
BImean				
NVmean	0.634	0.083	7.642	0.000
Identification	0.021	0.016	1.254	0.210
Older adults				
Identification				
Frequency	0.670	0.299	2.238	0.025
NVmean				
Frequency	0.119	0.064	1.857	0.063
Identification	0.063	0.018	3.403	0.001
BImean				
NVmean	0.568	0.088	6.470	0.000
Identification	-0.005	0.019	-0.283	0.777

Figure

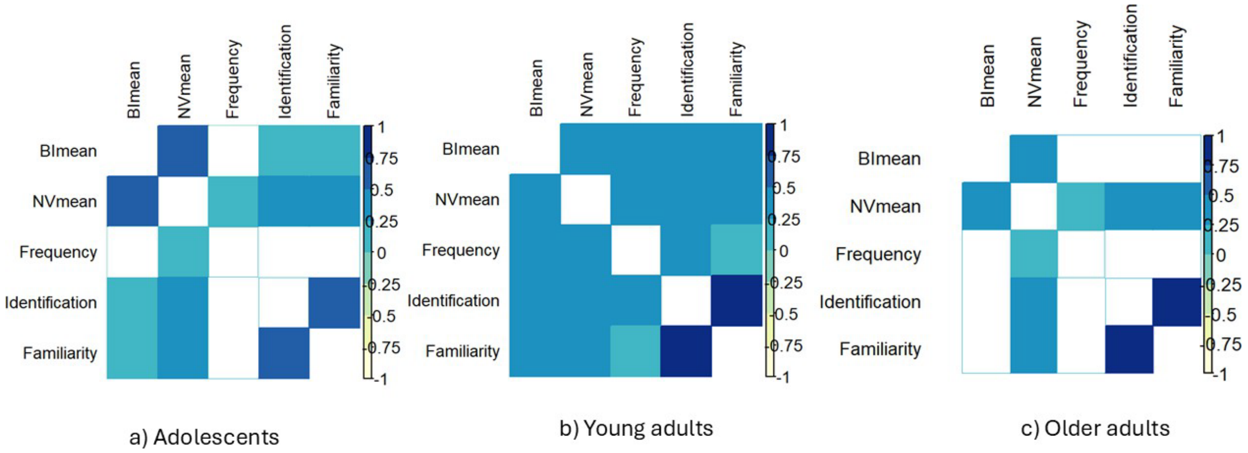

Figure S1: Correlation matrix heat map of Spearman's rho correlation coefficients among concepts for a) adolescents, b) young and c) older adults. PBI = Pro-conservation behaviour intentions, other abbreviations see Table A. The intensity of the blue colour indicates the strength of a positive correlation, with darker blues representing stronger positive correlations (closer to 1.00). Yellow colour would indicate negative correlations that are, however, not present in this heat map.
